# Statin Therapy for Primary and Secondary Prevention in Older Adults

**DOI:** 10.1007/s11883-024-01257-9

**Published:** 2024-11-25

**Authors:** Alicia Bao, Dean G. Karalis

**Affiliations:** Department of Cardiology, Jefferson University Hospital, Sidney Kimmel Medical College, 227 North Broad Street, Suite 200, Philadelphia, PA 19107 USA

**Keywords:** ASCVD prevention, Older adults, Statins, LDL cholesterol reduction

## Abstract

**Purpose of Review:**

Although statin therapy is well established to prevent atherosclerotic vascular disease (ASCVD) events in adults 40 to 75 years of age, it is less clear whether older adults benefit from statin therapy. The purpose of this review is to summarize the current evidence and guidelines on statin use for primary and secondary prevention in older patients.

**Recent Findings:**

Moderate to high intensity statin therapy decreases cardiovascular event rates in older patients with or at risk for ASCVD. Cardiac biomarkers and coronary calcium scoring can identify older patients at higher ASCVD risk who may benefit from statin therapy.

**Summary:**

Age alone should not be a deterrent to statin therapy in older patients. The decision to initiate statin therapy should occur after a patient to clinician discussion based on the patient's overall ASCVD risk and weighed against other clinical factors that influence the patient's life expectancy and quality of life.

## Introduction

Cardiovascular (CV) disease is the leading cause of death in both men and women in the United States (U.S.) and the prevalence of CV disease increases with age. According to data from the National Health and Nutrition Examination Survey 2017 to 2020 the prevalence of CV disease for men and women was 9.2 and 9.4% for those aged 40 to 59, 28.2 and 19.6% for those aged 60 to 79 and increased to 39.6 and 32.8% for those aged 80 years of older [1]. Older adults with a history of a myocardial infarction are also at increased risk of a recurrent CV event compared to younger individuals. In a large study of patients with an acute coronary syndrome a key risk factor for a recurrent CV event or CV death was age 65 years or older [2].

The population of the U.S. is growing and according to the 2020 Census, the U.S. population aged 65 or older grew five times faster than the total population. Although individuals 75 years of age or older grew at a slower rate, it is expected that the pace in the next decade will increase as baby boomers age into this group. In 2020 it was estimated that 25% of the U.S. population were 75 year of age or older [3]. As the population ages, clinicians will be faced with the challenges of treating and preventing CV disease in this aging population.

Statins remain the cornerstone of lipid lowering therapy for both primary and secondary prevention of atherosclerotic cardiovascular disease (ASCVD). The 2018 AHA/ACC/Multisociety cholesterol guideline as well as guidelines from Europe have provided recommendations for statin therapy for both primary and secondary prevention of ASCVD [4, 5]. The benefits of statin therapy are well established in patients with clinical ASCVD, and current guidelines recommend at least a moderate intensity statin for individuals over the age of 75. However, the benefits of statins for older patients without clinical ASCVD are less clear, and guidelines recommend an individualized approach after a patient to clinician discussion of the benefits and risks of statin therapy. The clinician caring for the older patient must weigh the individual’s CV risk against other factors such as the individual’s frailty, polypharmacy, their multiple comorbid conditions, any adverse effects that may occur from statin therapy, and the patient’s expected lifespan. This review will focus on the use of statin therapy in adults older than 75 years of age and assess the clinical evidence for statin use for primary and secondary prevention of CV disease, review statin safety in older adults, and provide practical suggestions on how to better assess CV risk in older adults without ASCVD who would benefit from statin therapy.

## Statin Therapy for Primary Prevention in Older Adults

Randomized clinical trials and meta-analyses have firmly established statin therapy for the primary prevention of ASCVD. Most clinical trials enrolled patients who were between the ages of 40 to 75 and there is less evidence of the benefits of statin therapy for primary prevention in older adults, particularly those greater than 75 years of age. However, some statin trials have enrolled patients over the age of 75.

The Antihypertensive and Lipid-Lowering Treatment to Prevent Heart Attack Trial – Lipid Lowering Trial (ALLHAT-LLT) compared 40 mg of pravastatin with usual care among 10,355 men and women aged 55 years of older with hypertension and at least one additional CV risk factor. Low density lipoprotein (LDL) cholesterol was reduced by 28% with pravastatin compared to 11% with usual care. After a mean follow-up of 4.8 years there was no difference observed between the pravastatin and usual care groups with respect to the primary endpoint of all-cause mortality or the secondary outcomes of nonfatal or fatal coronary heart disease events [6]. In a post-hoc analysis of participants 65 years or older there was no benefit of pravastatin in reducing CV events, while in those 75 years of age or older there was a nonsignificant trend toward increased all-cause mortality [7]. The lack of benefit seen in this trial may be related to the small difference in LDL cholesterol lowering between the groups. The PROspective Study of Pravastatin in the Elderly at Risk (PROSPER) trial, enrolled patients 70 to 82 years of age to evaluate the effects of statin therapy on both primary and secondary prevention of ASCVD events in an older population [8]. A total of 5804 men and women were randomized to 40 mg of pravastatin or placebo. Pravastatin lowered LDL cholesterol by 34% and in follow up of just over 3 years reduced the composite of coronary death, non-fatal myocardial infarction, and fatal and non-fatal stroke by 15% (hazard ratio 0.85, 95% CI 0.74–1.97, *p* = 0.014) and mortality from coronary disease by 24% (*p* = 0.043). However, the CV benefit was less in the subgroup of primary prevention older patients. This finding may be related to the short duration of the study and longer follow-up in the primary prevention group may have led to greater reductions in CV events.

More recent studies using higher intensity statin therapy have shown a benefit in reducing CV events in older patients. The JUPITER (Justification for Use of Statins in Prevention) trial randomized 17,802 healthy men and women with an LDL cholesterol less than 130 mg/dL and a high-sensitivity C-reactive protein (hs-CRP) of 2.0 mg/L or higher to rosuvastatin 20 mg daily or placebo [9]. LDL cholesterol was lowered to a median level of 55 mg/dL in the rosuvastatin group and in a secondary analysis of the 5695 individuals older than 70 years of age there was a 39% reduction in a composite of CV events (hazard ratio 0.61, 95% CI 0.46–0.82, *p* < 0.001) and a trend in reduction of all-cause death (hazard ratio 0.80, 95% CI 0.62–1.04, *p* = 0.09). The HOPE-3 trial randomized 12,705 individuals without known CV disease, but at intermediate CV risk, to rosuvastatin 10 mg daily or placebo [10]. Among older individuals with a mean age of 70 there was a 25% reduction in CV events. In a meta-analysis of the JUPITER and HOPE-3 trials, among patients aged 70 years or greater there was an overall significant 26% (hazard ratio 0.74, 95% CI 0.61–0.91. *p* = 0.0048) reduction in the risk of non-fatal myocardial infarction, non-fatal stroke, or CV death [11].

More recently, a study from Hong Kong investigated the benefits and risks of statin therapy for primary prevention in 69,981 older adults aged 75 to 84 years and 14,555 very old adults aged 85 years of age or older [12]. Patients were identified through electronic medical records, and propensity score matching was used to compare CV outcomes in patients who initiated versus those not initiating statin therapy. The primary endpoint was the cumulative incidence of major ASCVD events as defined by a composite of myocardial infarction, heart failure, stroke and all-cause death. Over an average follow-up of 5.3 years, the risk reduction for ASCVD events was found to be 1.2% in the intention-to-treat analysis and 5.0% in the per-protocol analysis in patients 75 to 84 years of age. In the patient population over the age of 85 years, the risk reduction was even more (4.4% in the intention-to-treat analysis and 12.5% in the per-protocol analysis). Although this study did not utilize true randomization, one of its strengths is its population-based approach, which allowed for a larger sample size. These results suggest that there may be a benefit to initiating statin therapy in older adults, even those older than 85 years of age.

Several meta-analyses have added further evidence for the benefit of statin therapy in older adults. The Cholesterol Treatment Trialists' (CTT) Collaboration performed an analysis of 28 trials (147,242 participants) comparing statin use versus control, as well as high intensity versus lower intensity statin use in adults over the age of 55 years of which 8% (14,483 participants) were older than 75 years of age [13]. Statin therapy or a more intensive statin regimen among patients with and without pre-existing vascular disease produced a 21% reduction in the risk of major vascular events defined as non-fatal myocardial infarction or stroke, coronary death, and coronary revascularization per 1.0 mmol/L (39 mg/dL) reduction in LDL cholesterol. However, among patients with no known vascular disease there was attenuation of the risk reduction observed with statin therapy with advancing age. In patients aged 75 years or older the risk of a major vascular event was reduced by 8% which did not reach statistical significance (95% CI, 0.73–1.16). The smaller risk reduction for primary prevention seen in this group of older patients may have been related to the limited sample size in the cohort of patients 75 years or older. These findings contrast with another meta-analysis of observational studies that compared statin use to non-use for primary prevention in patients over the age of 65 [14]. In this larger study of 815,667 patients, statin therapy was associated with a significantly lower risk of all-cause death (hazard ratio 0.86, 95% CI 0.79–0.93), CV death (hazard ratio 0.80, 95% CI 0.78–0.81), stroke (hazard ratio 0.85, 95% CI 0.76–0.94), and a trend to a lower risk of myocardial infarction (hazard ratio 0.74, 95% CI 0.53–1.02) [15]. The benefit of statin therapy in reducing all-cause death remained significant in patients over the age of 75.

The ongoing clinical trial of STAtin Therapy for Reducing Events in the Elderly (STAREE) is a placebo-controlled, randomized trial that is currently comparing atorvastatin 40 mg daily versus placebo in adults 70 years of age or older for primary prevention of ASCVD events and dementia [15]. As we await the results of this study, the evidence to date supports the use of statin therapy for primary prevention in select older patients at high CV risk.

## Picking the Right Older Patient for Statin Therapy

The 2018 AHA/ACC/Multisociety cholesterol guideline state that it may be reasonable (class 2b recommendation) to initiate moderate intensity statin therapy for individuals > 75 year of age who are considered at high risk of an ASCVD event [4]. Similarly, the 2021 European Society cholesterol guidelines list statin therapy as a class 2b recommendation for patients age > 70 [5]. Decisions to initiate statin therapy in this older age group should occur after a patient to clinician discussion considering the patient’s age, their ASCVD risk, risk of side effects from statin therapy, polypharmacy and drug to drug interactions, patient frailty, and the patients expected lifespan. The pooled cohort equation (PCE) is used to estimate the 10-year ASCVD risk of patients between the ages of 40 to 79, however as patients age, the factors (hypertension, diabetes, hypercholesteremia) used to calculate the PCE become increasingly prevalent, which lead to a tendency to overestimate actual 10-year ASCVD [16]. Additionally, age itself becomes a competing risk factor when considering the mortality benefit. For these reasons, additional variables that aid in risk stratification may be particularly beneficial for older patients. Cardiac imaging and biomarkers play a key role in assessing CV risk. They have been incorporated in current cholesterol guidelines and may aid the clinician in better assessing CV risk in the older patient and the decision to initiate statin therapy.

Several biomarkers have been described as predictors of CV events, with the most common being high sensitivity cardiac troponin T (cTnT), N-terminal pro-B-type natriuretic peptide (NT-proBNP), and hs-CRP. An elevated baseline cTnT, a marker of myocardial injury, may indicate subclinical myocardial ischemia, and is known to be associated with coronary artery disease and CV death in patients without known CV disease [17, 18]. Similarly, although a sensitive marker for congestive heart failure, elevated natriuretic peptide levels have been associated with increased risk of CV death even in patients without heart failure [19]. Notably, there was no association between NT-proBNP levels and coronary-specific health events. Finally, with the role inflammatory processes are thought to play in plaque stability, elevated levels of hs-CRP have been shown to be an independent risk factor for CV events, including myocardial infarction, ischemic stroke, and CV death [20, 21].

In addition to laboratory testing, imaging studies, such as non-contrast CT imaging to detect coronary artery calcium (CAC), carotid ultrasound to screen for atherosclerotic plaque, and measuring the ankle-brachial index, can detect subclinical atherosclerosis and serve as an indicator of ASCVD risk. The most validated imaging study is CAC scans, in which the deposition of calcium in atherosclerotic plaque on a CT scan can be quantified into a score that correlates with ASCVD risk. A CAC score of zero typically indicates a low risk of future ASCVD events in most populations. The presence of coronary artery calcium is a marker of advance underlying atherosclerosis and the higher the score the higher the ASCVD risk [22, 23]. The 2018 AHA/ACC cholesterol guidelines include a class IIb recommendation for using CAC scoring as a supplement to the decision to withhold or initiate statin therapy in patients older than 75 years of age [4]. Similarly, the 2021 European Society of Cardiology guidelines list CAC scoring as a class IIb consideration to improve risk classification [5].

Combining biomarkers with imaging studies have proven to be a useful assessment in predicting which intermediate risk patients may benefit from initiation of statin therapy. A pooled data of 16,581 patients found that abnormal cTnT, NT-proBNP, hs-CRP, and CAC score were each independently associated with increased risk of CV events; but moreover, with each additional abnormal test, the CV risk multiplied [24]. From this study, the authors devised a simple integer score counting the number of abnormal tests. A patient with only one abnormal test had a twofold increased risk of CV events, whereas a patient with all four abnormal tests had an eightfold increased risk.

Chemical biomarkers and imaging studies may be particularly useful tools in older adults to better define their CV risk and the benefit from statin therapy. The Atherosclerosis Risk in Communities (ARIC) study included over 15,000 individuals of a median age of 75.4 years and studied whether adding biomarkers in addition to a clinical assessment of their ASCVD risk factors improved risk prediction. All three biomarkers were associated with increased rates of ASCVD events over a short 4-year period, defined as coronary heart disease or stroke events, with a positive correlation in higher grading and higher rate. Addition of each biomarker into the PCE model also incrementally improved risk classification [25]. At present, the only biomarker recommended in the 2018 AHA/ACC/Multisociety cholesterol guidelines is hs-CRP [4]. However, measuring additional biomarkers may provide the clinician with a more complete assessment of the patient’s CV risk and whether they would derive benefit from statin therapy.

CAC scoring may prove most useful in determining which older patients would benefit from statin therapy. The BioImage Study included 5805 participants of mean age 69 years and found that 86% of the participants qualified for primary prevention statin therapy based on an ASCVD score ≥ 7.5%, but nearly one third of those individuals had no CAC on CT scanning [26]. The absence of CAC in an older adult can serve as a negative predictor of increased ASCVD risk, and thus increase specificity for atherosclerotic disease risk assessment. The 2018 AHA/ACC/Multisociety cholesterol guidelines includes a class IIb recommendation that downward reclassification may be considered in older patients aged 76 to 80 with a CAC score of zero, allowing the clinician to withhold statin therapy for primary prevention [4].

Based on observational studies, it would be reasonable for clinicians to consider elevated cardiac biomarkers as risk-enhancing factors in older patients that have other clinical risk factors for ASCVD. The absence of coronary calcium on imaging is a negative predictor of atherosclerotic disease and may be a useful tool in assessing CV risk in older patients in whom withholding initiation or de-prescribing statin therapy may be a reasonable choice.

## Statin Therapy for Secondary Prevention in Older Adults

Although the evidence for the use of statin therapy for primary prevention of ASCVD in older adults is mixed, the benefit of statin therapy for secondary prevention in older patients is well established. While not focused specifically on elderly patients, the Heart Protection Study was an early placebo-controlled trial that demonstrated a significant decrease in the incidence of major CV events across all age groups, including in the subgroup of patients aged 75 years and older. They found a decreased risk of vascular mortality, but not all-cause mortality, in older adults. Notably, the maximum age recruited in the study only reached 80 years. [27] The PROSPER trial was the first to specifically study older patients and included patients both with and without known ASCVD. A total of 2565 enrolled patients had a history of myocardial infarction, stable angina, or otherwise known coronary, peripheral, or cerebrovascular disease [8]. The study found that pravastatin had a greater risk reduction effect when used in these patients for secondary prevention (hazard ratio 0.78, 95% CI 0.66–0.93) compared to primary prevention (hazard ratio 0.94, 95% CI 0.77–1.15) for the primary endpoint of coronary heart disease death, or non-fatal myocardial infarction or stroke. Interaction testing showed no significant difference in characteristics between the patients of the two subgroups. Similarly, the CTT meta-analysis studied both primary and secondary prevention patients and found a larger percent risk reduction in major vascular events in older patients with existing atherosclerotic disease [13]. In fact, the proportional risk reduction in older patients with known vascular disease was like that of younger patients who were under the age 55 years. Among patients 70 years of age or older, only one-fifth of major vascular events occurred in those without known atherosclerotic disease. A large, secondary prevention meta-analysis pooled data from 9 randomized controlled trials and included 19,569 elderly patients over the age of 65 years with a history of prior myocardial infarction, known ASCVD, or stable angina [28]. This study found a 22% (relative risk 0.78; 95% CI 0.65 to 0.89) risk reduction in all-cause mortality, 30% (relative risk 0.70; 95% CI 0.53 to 0.83) reduction in coronary artery disease mortality, and 26% (relative risk 0.74; 95% CI 0.60 to 0.89) reduction in incidence of non-fatal myocardial infarction.

Based on current available data from randomized controlled trials the 2018 AHA/ACC/Multisociety cholesterol guidelines provide a recommendation for initiation of at least moderate-intensity statin therapy in adults > 75 years of age with known ASCVD [4]. There appears to be an increased benefit of more intensive LDL cholesterol lowering even in elderly patients [29] with higher-intensity statin; however, this benefit could be outweighed by side effects that come with higher doses. For patients who were previously prescribed and already tolerating a high-intensity statin therapy, the guidelines include a class IIa recommendation to continue the high-intensity dose. Notably, for patients with very high risk ASCVD, i.e., those with a history of major CV event such as prior acute coronary syndrome or myocardial infarction, ischemic stroke, or symptomatic peripheral arterial disease, as well as other CV risk factors, there is no longer a distinction of statin intensity by age. High-intensity or maximally tolerated statin therapy is advised for all patients in this very high risk group, as well as additional statin-lowering therapy (i.e., ezetimibe, bempedoic acid or PCSK-9 inhibitors) if LDL cholesterol remains ≥ 70 on maximum statin dosing.

## Statin Side Effects

Studies have demonstrated that higher-intensity statin therapy leads to significantly more risk reduction in CV events compared to low or moderate-intensity statin therapy in patients of all ages, including those over the age of 75 [29]; however, this benefit must be considered alongside the side effect profile that can be particularly harmful to older individuals. The most common side effect of statins is statin-associated muscle symptoms (SAMS), manifesting as subjective myalgia in the absence of increased creatine kinase (CK) levels. Although typically not dangerous, SAMS is frequently cited as the reason for discontinuing statin therapy. In elderly adults, frailty and risk of falls may be a concerning sequela of myalgias. Fortunately, more serious adverse events that have been reported with statin therapy such as myositis or rhabdomyolysis, are rare even among elderly patients.

Although not a major study endpoint, adverse events were briefly highlighted in most of the previously discussed clinical trials. In the ALLHAT trial, almost 80% of patients who were assigned pravastatin were still taking the medication at 6-year follow-up. Roughly half of patients did not cite a reason for discontinuation, but the other half of those patients cited side effects as the reasons for discontinuation [7]. In the JUPITER and HOPE-3 trials, the rate of medication discontinuation was higher among individuals over the age of 70 years, although the reasons for discontinuation were not reported [10]. The PROSPER trial demonstrated similar rates of serious adverse events in both the pravastatin and control group, with no reported incidences of rhabdomyolysis or elevated creatine kinase levels above 10 times the upper limit of normal [8]. Only one patient in each group experienced significantly elevated transaminases. An increased incidence of cancer was noted in the pravastatin group, with the majority being gastrointestinal (*p* = 0.020). However, the authors performed a small meta-analysis of other studies utilizing pravastatin and did not find an association with increased cancer rate once combined with the larger patient sample.

The Provider Assessment of Lipid Management (PALM) registry was a cross-sectional survey that included 1704 patients > age 75 years who had an indication for statin initiation after omitting age criteria in the PCE [30]. When compared to the cohort under age 75, older patients significantly reported fewer statin side effect (*p* = 0.003) or myalgias (*p* < 0.001). The prevalence of discontinuation was similar between the groups, and quoted reasons for discontinuation were not statistically different. However, other studies have noted differing results. In the Incremental DEcrease through Aggressive Lipid Lowering (IDEAL]) trial, the safety of high-intensity atorvastatin (80 mg) versus moderate intensity simvastatin (20 to 40 mg) was compared in patients age < 65 years and ≥ 65 years [31]. The older group reported significantly more patient-reported adverse events compared to the younger group. Notably, reported cases of rhabdomyolysis and elevated transaminases were exceedingly rare in both groups. There was no difference in rates of adverse event reporting between the high-intensity and moderate-intensity groups, although more patients taking the 80 mg atorvastatin dose experienced elevated transaminase levels. A meta-analysis performed across 13 randomized controlled trials with over 19,000 participants found the risk of incident diabetes to be higher in patients assigned to statin therapy, particularly in studies that enrolled older patient populations [32]. The authors acknowledge that more research needs to be done to investigate whether this is a true effect or due to residual confounding variables. The incidence of new onset diabetes mellitus with statin therapy is overall low and most likely to occur with high-intensity statin doses and in patients with pre-diabetes.

Overall, statin therapy is safe and well tolerated in older patients, However, older patients have more co-morbidities and are at risk for drug to drug interactions. An ambulatory study using the DRUG-REAX database found a significantly increased incidence of clinically relevant drug-drug interaction in patients over age 75 years [33]. The most common drug interactions were with amiodarone, diltiazem, verapamil, and digoxin. Several of these drugs are CYP3A4 inhibitors, the same enzyme that metabolizes simvastatin and atorvastatin. A systematic review of other studies found a wide variety of drug-drug interaction incidences, ranging from as low as 0.19% to 33%, and noted inconsistencies in the definition of a clinically relevant interaction. [34] More studies need to be done to examine whether these drug interactions confer increased risk of serious adverse effects and clinically significant side effects in older patients.

## Conclusions

**Fig. 1:**
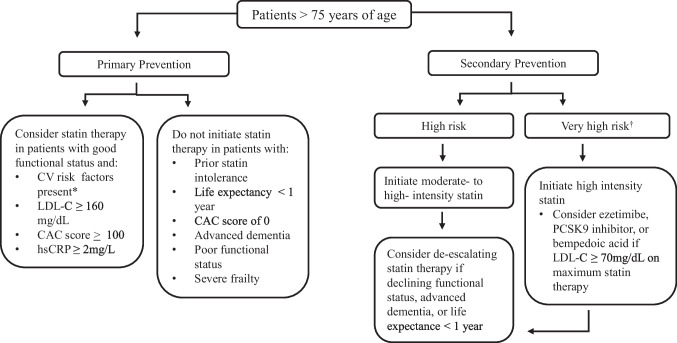
*CV risk factors: type 2 diabetes, active smoking, uncontrolled hypertension, chronic kidney disease †Very high-risk patients include those with recent ACS within the past year, history of myocardial infarction, history of ischemic stroke, and symptomatic peripheral arterial disease plus other CV risk factors. Adapted from the 2018 AHA/ACC/Multisociety guideline [4]. Abbreviations: CV = cardiovascular; LDL-C = low-density lipoprotein cholesterol; CAC = coronary artery calcium; hsCRP = high-sensitivity C-reactive protein

As the elderly patient population of the U.S. grows, clinicians will increasingly face the decision of whether their patients may benefit from taking a preventative statin. Many considerations including the perceived benefit, anticipated lifespan, competing co-morbidities, and overall functional status need to be weighed (Figure 1). The GERIATRIC 5Ms should be applied as a framework to remind clinicians that the Mind, Mobility, Medications, Multicomplexity, and Matters of the patient should be discussed in a shared decision making process prior to initiation of statin therapy [35]. Based on the current AHA/ACC/Multisociety guideline, it may be reasonable for physicians to consider initiation of moderate-intensity statin therapy in high-risk patients over age > 75 years. The PROSPER randomized controlled trial has demonstrated the benefit on CV events and mortality in elderly patients when used for secondary prevention, but the data for patients solely with risk factors but no clinical ASCVD is currently under investigation. Supplemental testing may prove useful in determining which patients are at high risk. The degree of elevation in cardiac biomarkers appears to be proportional to the risk of CV events and may indicate an increased benefit in statin therapy as does the presence of advanced subclinical atherosclerosis. Conversely, a patient with a CAC score of zero has a low 10-year ASCVD risk, and thus may not require a statin even if other risk factors are present. This review focused on statin therapy specifically and does not address other non-statin LDL cholesterol lowering drug classes, such as ezetimibe, bempedoic acid, or PCSK-9 inhibitors, which could be considered in older patients at very high ASCVD risk who require additional LDL cholesterol lowering on a maximally tolerated statin. While we await the results of ongoing statin trials in older patients, statin prescribing should remain a shared decision between patient and provider, but overall, the clinical evidence appears to indicate that statins are a safe and beneficial medication to reduce ASCVD risk in an older population.

## Key References

- Grundy SM, Stone NJ, Bailey AL, Beam C, Birtcher KK, Blumenthal RS, et al. 2018 AHA/ACC/AACVPR/AAPA/ABC/ACPM/ADA/AGS/APhA/ASPC/NLA/PCNA Guideline on the management of blood cholesterol: report of the American College of Cardiology/American Heart Association task force on clinical practice guidelines. Circulation 2019; 139: e1082-e1143. https://doi.org/10.1161/CIR.0000000000000625.
  - Most recent guidelines for the American Heart Association, American College of Cardiology, and other societies for the use of statin therapy in older patients.
- Cholesterol Treatment Trialists’ Collaboration. Efficacy and safety of statin therapy in older people: a meta-analysis of individual participant data from 28 randomized controlled trials. The Lancet 2019; 393: 407–415. https://doi.org/10.1016/S0140-6736(18)31942-1.
  - Meta-analysis analyzing over 14,000 older patients in 28 clinical trials that showed definite benefit of secondary prevention statin in older adults but decreasing benefit with increasing age for primary prevention.
- Awad K, Mohammed M, Zaki MM, et al. Association of statin use in older people primary prevention group with risk of cardiovascular events and mortality: a systematic review and meta-analysis of observation studies. BMC Medicine 2021; 19: 139. https://doi.org/10.1186/s12916-021-02009-1.
  - A large meta-analysis that showed statin therapy significantly reduced major cardiovascular events and cardiovascular death in older primary prevention patients.
- Gore MO, Ayers CR, Khera A, et al. Combining biomarkers and imaging for short‐term assessment of cardiovascular disease risk in apparently healthy adults. J Am Heart Assoc. 2020–08-04 2020;9(15):e015410. https://doi.org/10.1161/jaha.119.015410.
  - Demonstrated that risk of major cardiovascular events multiplied with each additional positive biomarker (c-TNT, NT-proBNP, hs-CRP) or the presence of subclinical coronary artery calcium.
